# Rex in *Caldicellulosiruptor bescii*: Novel regulon members and its effect on the production of ethanol and overflow metabolites

**DOI:** 10.1002/mbo3.639

**Published:** 2018-05-23

**Authors:** Kyle Sander, Daehwan Chung, Doug Hyatt, Janet Westpheling, Dawn M. Klingeman, Miguel Rodriguez, Nancy L. Engle, Timothy J. Tschaplinski, Brian H. Davison, Steven D. Brown

**Affiliations:** ^1^ Department of Chemical and Biomolecular Engineering University of Tennessee Knoxville Tennessee; ^2^ Bredesen Center for Interdisciplinary Graduate Research and Education University of Tennessee Knoxville Tennessee; ^3^ BioEnergy Sciences Center Oak Ridge National Laboratory Oak Ridge Tennessee; ^4^ Department of Genetics University of Georgia Athens Georgia; ^5^ Biosciences Division Oak Ridge National Laboratory Oak Ridge Tennessee; ^6^ Present address: National Renewable Energy Laboratory Golden CO; ^7^ Present address: LanzaTech Skokie IL

**Keywords:** *Caldicellulosiruptor bescii*, consolidated bioprocessing, ethanol, Rex

## Abstract

Rex is a global redox‐sensing transcription factor that senses and responds to the intracellular [NADH]/[NAD ^+^] ratio to regulate genes for central metabolism, and a variety of metabolic processes in Gram‐positive bacteria. We decipher and validate four new members of the Rex regulon in *Caldicellulosiruptor bescii*; a gene encoding a class V aminotransferase, the HydG FeFe Hydrogenase maturation protein, an oxidoreductase, and a gene encoding a hypothetical protein. Structural genes for the NiFe and FeFe hydrogenases, pyruvate:ferredoxin oxidoreductase, as well as the *rex* gene itself are also members of this regulon, as has been predicted previously in different organisms. A *C. bescii rex* deletion strain constructed in an ethanol‐producing strain made 54% more ethanol (0.16 mmol/L) than its genetic parent after 36 hr of fermentation, though only under nitrogen limited conditions. Metabolomic interrogation shows this *rex‐*deficient ethanol‐producing strain synthesizes other reduced overflow metabolism products likely in response to more reduced intracellular redox conditions and the accumulation of pyruvate. These results suggest ethanol production is strongly dependent on the native intracellular redox state in *C. bescii*, and highlight the combined promise of using this gene and manipulation of culture conditions to yield strains capable of producing ethanol at higher yields and final titer.

## 1 INTRODUCTION

Liquid transportation fuel demand is projected to increase through 2075 (Fulton, Lynd, Körner, Greene, & Tonachel, 2015) and an inexpensive, reliable way to produce bioethanol from lignocellulosic feedstocks will be necessary to meet increased demand. Consolidated bioprocessing (CBP) is expected to be a cost‐saving way for producing bioethanol (Lynd, van Zyl, McBride, & Laser, 2005), primarily because of its use of inexpensive lignocellulosic feedstocks and lower capital and operating costs due to biocatalysts that produce enzymes for the deconstruction and solubilization of feedstocks to soluble carbohydrates.

*Caldicellulosiruptor bescii* is a promising candidate biocatalyst for this single‐step ethanol production process (Yang et al., 2010). The genus *Caldicellulosiruptor* are anaerobic hyperthermophiles, which can ferment a variety of organic substrates (Hamilton‐Brehm et al., 2010), and produce nearly theoretical amounts of hydrogen in order to recycle redox cofactors (Bielen, Verhaart, van der Oost, & Kengen, 2013; van de Werken et al., 2008). It is capable of solubilizing lignocellulosic biomass through the activity of its suite of CAZymes (Blumer‐Schuette et al., 2012, 2015; Brunecky et al., 2013), and fermenting many of the resulting soluble carbohydrates, producing primarily acetate, lactate, hydrogen, and CO_2_. Some species can also produce ethanol, though this trait is not conserved across the genus. *C. bescii* does not natively produce ethanol as a fermentation product, but was recently engineered to produce ethanol directly from lignocellulosic biomass substrates by introducing an *adhE* gene, which is constitutively expressed (Chung, Cha, Guss, & Westpheling, 2014; Chung et al., 2015).

Ethanol synthesis in *C. bescii* relies on cofactors from the organism's native redox system, as is the case in other CBP organisms (Biswas, Zheng, Olson, Lynd, & Guss, 2015; Li et al., 2012). These redox systems are plastic and subject to modulation through genetic modifications or by altering growth conditions. Eliminating lactate production in *C. bescii* increased overall hydrogen production (Cha, Chung, Elkins, Guss, & Westpheling, 2013), while eliminating the NiFe membrane‐bound hydrogenase decreased ethanol yield in a strain expressing an exogenous bifunctional AdhE (Cha, Chung, & Westpheling, 2015). Another method of redox modulation by *Caldicellulosiruptor* is demonstrated by the closely related bacterium *Caldicellulosiruptor saccharolyticus. C. saccharolyticus* produces lactate upon sparging a continuously growing culture with hydrogen (Bielen et al., 2013). It was suggested that this results from the introduced hydrogen inhibits hydrogen generation by hydrogenases, resulting in NADH‐driven metabolic forcing of lactate dehydrogenase activity. While *Caldicellulosiruptor* species rely heavily on hydrogenases and fermentation to facilitate redox balance, other metabolic components also contribute to its redox metabolism. *C. bescii* strains expressing exogenous alcohol dehydrogenases with different redox cofactor requirements synthesized variable amounts of ethanol when grown under similar conditions (Chung et al., 2015). These experiments demonstrate that a better understanding of cellular redox systems is needed to more effectively engineer this biocatalyst to produce ethanol more effectively.

Rex is a well characterized and conserved global redox responsive transcription factor (Ravcheev et al., 2012) that was first characterized in *S. coelicolor* as a global repressor able to detect and regulate gene expression in response to the intracellular [NADH]/[NAD^+^] couple (Sickmier et al., 2005). In doing so, Rex not only regulates the poise of this redox couple, but the cellular redox state in general, as evidenced by its conserved transcriptional regulation of redox genes that are not NAD(H) dependent (Ravcheev et al., 2012). Conserved Rex regulon members include genes involved in energy conversion, redox metabolism, glycolytic and fermentation genes, and NAD biosynthesis (Ravcheev et al., 2012). Examples of genes where Rex regulation is found to be taxon specific and less conserved are those of hydrogenases, heme biosynthesis, sulfate reduction (Christensen et al., 2015) and biofilm formation (Bitoun, Nguyen, Fan, Burne, & Wen, 2011), solventogenic metabolism (Zhang et al., 2014), nitrate and chlorate metabolism/resistance (Carlson et al., 2015), and cytochrome biosynthesis (Larsson, Rogstam, & von Wachenfeldt, 2005).

Redox sensing and regulation by the transcriptional regulator Rex in the closely related species *Caldicellulosiruptor saccharolyticus* was partially inferred from a differential gene expression study of cells grown under hydrogen sparging (Bielen et al., 2013). Genes encoding elements of both the NiFe and FeFe hydrogenases were predicted to be under transcriptional control of Rex, as were the subunits of pyruvate:ferredoxin oxidoreductase. Lactate dehydrogenase is not predicted to be controlled by Rex in *C. saccharolyticus* (Bielen et al., 2013), though it is predicted to be controlled by Rex in other many other organisms (Ravcheev et al., 2012). Rex is predicted to be a global repressor in *C. saccharolyticus*, regulating expression of other regulatory elements such as a histidine kinase, a CopG family transcription factor, the iron uptake regulator Fur, as well as the *rex* gene itself. Other regulatory targets of Rex and action of some regulatory elements in the regulon of *C. saccharolyticus* remain unknown and unexplored. A consensus‐binding sequence for Rex was predicted in *C. saccharolyticus* (Bielen et al., 2013) that is similar to predicted Rex‐binding sequences identified in bacteria from other genera (Novichkov et al., 2013). The operator site in *C. saccharolyticus* and other organisms is an 18–20 bp palindrome sequence with the overall consensus sequence of TTGTGAANNNNTTCACAA (Ravcheev et al., 2012). The residues of the binding sequence important for Rex‐DNA interaction have been shown to be its most conserved residues (Brekasis & Paget, 2003; McLaughlin et al., 2010; Pagels et al., 2010; Pei et al., 2011).

Deleting the *rex* gene can have dramatic effects on intracellular redox state and the production of ethanol. A strain of *Clostridium acetobutylicum* containing a disrupted *rex* gene produced more ethanol and butanol after 60 hr than its parent strain or a rex deletion strain complemented with the *C. acetobutylicum rex* gene (Wietzke & Bahl, 2012). Derepression of *adhE* genes, which are transcriptionally controlled by Rex in *C. acetobutylicum* (Wietzke & Bahl, 2012; Zhang et al., 2014), and increased NADH‐dependent AdhE activity were shown to coincide with increases in ethanol and butanol production.

The goal of this study is to understand the redox metabolism in this unique and biotechnologically relevant species and genus, specifically as it pertains to ethanol synthesis, and we do so by studying the *rex* gene in *C. bescii*. A comprehensive understanding of redox metabolism in *C. bescii* will allow more effective engineering of redox systems to promote synthesis of ethanol at higher yield and productivity.

## 2 EXPERIMENTAL PROCEDURES

### 2.1 Batch growth and fermentation

*C. bescii* was grown in 50 ml culture volumes in sealed 135 ml serum bottles. The medium was comprised of components as described previously (Farkas et al., 2013), with maltose being used as the primary carbon source, and ammonium chloride as the primary nitrogen source. Media were prepared, adjusted to pH of 6.8, and allowed to become anaerobic overnight through dissolved oxygen exchange in an anaerobic chamber containing an environment of 5% H_2_, 10% CO_2_, and the balance N_2_. Cultures were grown at 75°C shaking at 200 rpm. Samples for cell growth were collected as 1 ml aliquots and measured for absorbance at OD_680_. Nitrogen limited batch growth was conducted as outlined above with the only difference being the final concentration of ammonium chloride (the only source of reduced nitrogen in LOD media) of 0.467 mmol/L rather than 4.67 mmol/L as in standard LOD media.

### 2.2 pH controlled fermentation

Cells were cultured in 3L Applikon Ez‐Control fermenters (Applikon Biotechnology, Delft, Netherlands) in a working culture volume of 1.5 L at a growth temperature of 75°C. Maltose, resazurin, and an appropriate volume of water were added to assembled fermenters and autoclaved. Fermenters were cooled while sparging with N_2_ gas. Upon cooling, other media components were added as presterilized stock solutions. The media was again heated and sparged with N_2_ gas to ensure anaerobic conditions. Upon reaching 75°C, the pH was adjusted to 7.1 by sparging with an 80%/20% N_2_/CO_2_ gas mix. The pH was then aseptically checked using a second probe which had been calibrated with fresh pH buffers maintained at 75°C. Any differences were accounted for as the pH probe offset. Fermenters were inoculated to equivalent OD_680_ of 0.01–0.05 with batch grown cultures grown to mid‐log phase. Stirring was maintained at 200 rpm without gas being sparged during growth, though the headspace outlet line was kept open to allow fermentation off‐gasses to vent through a sterilized water trap.

### 2.3 Fermentation product analysis

Samples were collected from batch serum bottles or fermenters as 1 ml aliquots and centrifuged at maximum speed in a microcentrifuge for 5 min, followed by collecting and filtering (0.22 μm) the supernatant. 250 μl of filtered supernatant was added to 1.75 μl of 2 mol/L H_2_SO_4_ and 20 μl of this mixture was injected onto a Biorad Aminex 87H column operated on a Hitachi LaChrom Ultra HPLC system (Hitachi High Technologies America, Dallas, TX). Chromatograms were collected on a Hitachi RI detector (part number L‐2490).

### 2.4 Mutant construction

*C. bescii rex* deletion mutants were constructed as described previously (Chung, Farkas, Huddleston, Olivar, & Westpheling, 2012). Strains generated for this study, necessary oligonucleotides, and plasmids used for strain generation are listed in Table 1. Briefly, an integrating suicide vector was prepared containing 1,000 bp of homology overlap to genomic sequence immediately 5′ and 3′ of the *rex* (ATHE_RS03255) coding sequence (Figure S1a). Plasmids were transformed into the respective genetic background, either strain JWCB005 (Chung, Cha, Farkas, & Westpheling, 2013) or strain JWCB032 (Chung et al., 2014), and transformants selected in LOD media containing no uracil to enforce prototrophic growth and plasmid integration. These cultures were subsequently plated on LOD media containing 40 μmol/L uracil (to allow for a return to strain auxotrophy) and 6 mmol/L 5‐fluororotic acid (Oakwood Products Inc., Estill, South Carolina) to screen and select for the double homologous recombination event. Deletions were confirmed by PCR (Figure S1b) and Sanger sequencing of the *rex* gene genomic locus.

**Table 1:** Primers, plasmids, and *C. bescii* strains generated and/or used in this study

(A)	
Primer name	Sequence
pDCW88 gib assy backbone fwd	gtgcactctgacgctc
pDCW88 gib assy backbone rev	ggtaccaccagcctaac
pDCW88_athe_0654_up_fwd	tccaatgatcgaagttaggctggtggtaccatatcttcaattttgtccacagcag
pDCW88_athe_0654_up_rev	ttacataacgcattcatttcacctcaagtccttttctcccccttatcttcttttg
pDCW88_athe_0654_down_fwd	gacttgaggtgaaatgaatgc
pDCW88_athe_0654_down_rev	gttttcgttccactgagcgtcagagtgcacaacctttctaaattacttgcaacaag
upstm 5′ flank fwd athe_0654_P3	agaatattgaagcgccgaac
dnstm 3′ flank rev athe_0654_P3	gtggaaaaatcaccccagaa
internal fwd athe_0654_P3	gggtttggtcagcaaggata
internal rev athe_0654_P3	acccttaatcccaccttcaa
3′ flank rev seq athe_0654_P3	tttgcaagatttgcgtaaga

(B)		
Plasmid	Description	Reference
pETE01	Non‐replicating suicide vector used to generate strains JWCB005Δ*rex* and JWCB032Δ*rex*	This study
pTXB1::*rex*	IPTG‐inducible expression vector used to express recombinant Rex protein in *E. coli* T7 Express	This study

(C)			
**Strains**	**Description**	**Genotype**	**Reference**
JWCB005	Genetic background strain used to generate JWCB005Δ*rex*, wildtype *ldh* locus	Δ*pyrFA* (*ura* ^−^/5‐FOA^R^)	Farkas et al. (2013)
JWCB005 Δ*rex*	Markerless *rex* deletion using strain JWCB005 as genetic parent	Δ*pyrFA* Δ*rex* (*ura* ^−^/5‐FOA^R^)	This study
JWCB032	Genetic background strain used to generate JWCB032Δ*rex*,* ldh* locus disrupted by ISCbe4, expressing genomically integrated bifunctional alcohol dehydrogenase (AdhE) from *C. thermocellum*	Δ*pyrFA ldh*::ISCbe4 Δ*cbeI*::Ps‐layer Cthe‐*adhE (ura* ^−/^5‐FOA^R^)	Hamilton‐Brehm et al. (2010)
JWCB032 Δ*rex*	Markerless *rex* deletion using strain JWCB032 as genetic parent	Δ*pyrFA* Δ*rex ldh*::ISCbe4 Δ*cbeI*::Ps‐layer Cthe‐*adhE* (*ura* ^−^/5‐FOA^R^)	This study

### 2.5 RNA‐seq analysis

Samples were collected from fermenter‐grown cultures of JWCB005Δ*rex* and its parent strain JWCB005 at early, mid, and late log phase (Figure S2a). Thereafter, 25 ml culture aliquots were harvested, centrifuged at 20,000*g* in a Piramoon fixed angle FiberLite rotor (ThermoScientific, Waltham, MA) for 4 min at 4°C, decanted, snap frozen in liquid nitrogen, and stored at −80°C. Total RNA was extracted by first incubating cell pellets in 250 μl of 20 mg/ml Lysozyme (Sigma Aldrich part number L‐7651, St. Louis, MO) resuspended in SET buffer (50 mmol/L Tris‐HCl pH 8.0 50 mmol/L EDTA, 20% w/v Sucrose) and incubated in a dry stationary bath at 37°C for 8 min, vortexing briefly every 2 min. RNA was purified with a Qiagen RNEasy Kit according to manufacturer's protocol (Qiagen, Hilden, Germany). RNA concentration was quantified with a Nanodrop 1000 instrument (ThermoScientific) and RNA quality was assessed via RNA Integrity Numbers (RIN) obtained with an Agilent 2100 Bioanalyzer and corresponding RNAchip (Agilent Technologies, Santa Clara, CA). Ribosomal RNA was then depleted from total RNA samples with a RiboZero rRNA Removal Kit (Illumina Inc. San Diego, CA) following manufacturer's instructions. Next, cDNA was synthesized from RNA depleted of ribosomal RNA with a TruSeq Stranded mRNA Library Preparation Kit (Illumina Inc) following the manufacturer's protocol. The cDNA libraries were sequenced on an Illumina Hi‐seq 2500 using v4 chemistry (Illumina Inc.) and demultiplexed as a sequencing service provided by The Genomic Services Lab at HudsonAlpha Institute for Biotechnology (HudsonAlpha, Huntsville, AL). Each sample library was sequenced on two different sequencing lanes and reads containing identical barcodes from each lane were combined for subsequent analysis. Reads obtained were scored for quality, trimmed, mapped, and the mapped reads counted with the corresponding functions in the CLC Genomics Workbench version 8 using default settings for genome analysis of prokaryotes. Raw read counts for each coding sequence were used as input for differential expression analysis, using the DEseq2 (Love, Huber, & Anders, 2014) package as part of the Bioconductor Suite in R. Genes were considered differentially expressed if they displayed differential normalized log_2_‐transformed read count abundance >0.5 or <−0.5 with a Benjamini‐Hochburg adjusted *p* < .05.

### 2.6 Metabolomic profiling

Differential metabolomic profiling was conducted on strains JWCB032Δ*rex* and JWCB032 collected after 36 hr of batch serum‐bottle growth in 50 ml culture volume of nitrogen‐limiting media, as described above. Here, 50 ml replicates were collected, centrifuged, and snap frozen as described in the sample collection section of RNA‐seq analysis. Additionally, following centrifugation, the supernatants were aliquoted separately, snap frozen, and stored at −80°C for metabolomic analysis. Cell biomass was pooled from three 50 ml cultures to make one replicate for intracellular metabolite analysis, as it was needed to attain sufficient signal intensity. Cell pellets and supernatants were analyzed for intracellular and extracellular metabolites, respectively, as described previously (Holwerda et al., 2014).

### 2.7 Transcription factor‐binding site prediction

The consensus Rex‐binding sequence identified in *C. saccharolyticus* (Bielen et al., 2013) was used to seed a search genomic regions 300 bp upstream of every coding DNA sequence in the *C. bescii* genome. Binding sites were identified and scored based on similarity to this consensus‐binding site, yielding 63 total putative Rex‐binding sites across the *C. bescii* genome (Table S1). Homology scores for these sites ranged from 8.75 (least homologous) to 10.5 (most homologous).

### 2.8 *Rex* Protein purification

Rex protein was purified by first expressing the *C. bescii rex* coding sequence on plasmid pTXB1 (New England Biolabs part number N6707S, Ipswitch, MA) upstream of the gyrase intein and chitin‐binding domain (CBD), yielding plasmid pTSB1::*rex* (Table 1B). This plasmid was transformed into T7 Express *E. coli* cells (New England Biolabs). Cells were grown, induced and harvested according to manufacturer's suggested instructions. Recombinant Rex (rRex) protein was purified from induced cell biomass according to protocols supplied with the IMPACT protein purification kit (New England Biolabs).

### 2.9 Electromobility shift assays

Electromobility shift assays were carried out to test Rex binding to predicted operator sites listed in Table 2. Probes used were biotin‐labeled 50‐mers of double stranded DNA consisting of the 18 bp putative‐binding site centered on the probe and surrounding genomic sequence on either side to make 50 bp. Probes were ordered as complimentary oligonucleotides, with one oligonucleotide biotin labeled (Integrated DNA Technologies, Coralville, IA). All probes were annealed in a mixture containing 3 μl of 100 μmol/L of each oligonucleotide, 3 μl of 10× polynucleotide kinase buffer (Roche part number 12579400, Roche Holding AG, Basel, Switzerland), 3 μl of 0.5 mol/L NaCl, 18 μl of DEPC treated H_2_0. Mixtures were heated at 96°C for 5 min in a Fisher Scientific Isotemp Stationary Bath (ThermoScientific) after which the heat block was removed from the bath and placed on a lab bench for 60 min to cool slowly. Probes were then purified using a Qiagen PCR clean‐up kit (Qiagen) according to manufacturer's instructions.

**Table 2:** Rex operator‐binding sites chosen for in vitro binding validation from predicted Rex operator sites in the *C. bescii* genome

Locus tag downstream of operator site	Predicted transcription unit	Distance from ATG (bp)	RegPrecise (Novichkov et al., 2013) Predicted Regulon	Predicted Regulon in (Bielen et al., 2013)	Site Homology Prediction Score (This study)	log_2_ (JWCB005Δrex/JWCB005) of downstream gene		
						Early Log Phase	Mid Log Phase	Late Log Phase
ATHE_RS00825, Athe_0168	2 genes; CopG family transcriptional regulator, HydG hydrogenase maturation protein	181	X	X	10.5	−0.21	−0.23	−0.27
ATHE_RS03255, Athe_0654	1 gene; Rex	41	X	X	9.25	N/A	N/A	N/A
ATHE_RS04105, Athe_0820	2 genes, Ferredoxin, ‘XOR’ oxidoreductase protein (Scott et al., 2015)	148			9.5	0.86	0.60	0.75
ATHE_RS04125, Athe_0824	3 genes; tungsten transport system (Scott et al., 2015)	274			8.75	0.47	0.38	0.77
ATHE_RS04390, Athe_0874	4 genes; subunits of pyruvate/ketoisovalerate:ferrodoxin oxidoreductase	112		X	9.5	−0.28	−0.73	−0.62
ATHE_RS04720, Athe_0942	2 genes; hypothetical protein, hypothetical protein	40			10	−1.46	−1.42	−0.83
ATHE_RS05415, Athe_1082	19 genes; Ech hydrogenase system	147	X	X	9	−0.43	−0.80	−1.13
ATHE_RS06475, Athe_1295	5 genes; Hyd hydrogenase system	39	X	X	10	−0.13	−0.35	−0.38
ATHE_RS10680, Athe_2126	3 genes; class V aminotransferase, phospoglycerate dehydrogenase (NADH), hypothetical protein	88			8.75	0.59	0.63	0.57
ATHE_RS11210, Athe_2226	1 gene; pyruvate carboxyltransferase (KEGG) or 2‐isopropylmalate synthase LeuA (RefSeq)	169		X	8.75	0.08	0.12	0.22

Sites were chosen from homologous sites identified in the RegPrecise (Novichkov et al., 2013) curated regulon, previously inferred Rex‐binding sites in *C. saccharolyticus* (Bielen et al., 2013), and observed differential expression between JWCB005Δ*rex* and its parent strain (JWCB005). Gray shading indicates nonsignificant values. Black values indicate values which are significant (Benjamini‐Hochburg adjusted *p* > 0.05). Putatively regulated transcriptional unit operon structure are reported as predicted in the DOOR database (Mao et al., 2014) and previous literature (Scott et al., 2015; van de Werken et al., 2008).

Electromobility shift assay reactions were conducted in 20 μl volumes and the following components were added in the following order: 1× EMSA‐binding buffer (LightShift Chemiluminescent EMSA Kit part number 20148, ThermoScientific), 25 μg dI‐dC, recombinant Rex protein in various concentrations noted on figures, and 0.1 nmol/L biotin‐labeled 50 bp dsDNA probes. In reactions containing NADH or NAD^+^, these components were added from freshly prepared stocks kept on ice prior to adding recombinant Rex protein but after adding other components. Reactions were incubated for 30 min at room temperature after which 5 μl of EMSA loading buffer containing 15% w/v Ficoll (Sigma Aldrich, St. Louis, MO) and 0.4% w/v Orange G (Sigma Aldrich) was added to each reaction. 20 μl of the reaction mixture containing loading buffer was loaded onto a Novex 6% DNA Retardation Gel (Invitrogen # EC63652BOX, Life Technologies Corporation, Carlsbad, CA) and run in 0.5× TBE (Invitrogen # LC6675, Life Technologies Corporation) in an Invitrogen Novex Mini‐Cell X Cell SureLock (Life Technologies Corporation) at 90 V for 45 min, or until Orange G from gel loading buffer had run ¾ of the length of the gel. After electrophoresis products were transferred onto Biodyne B Pre‐Cut Modified Nylon 0.45 μm Membranes (Product # 77016 ThermoScientific) using the Invitrogen X Cell Blot Module (catalog # EI9051) and Invitrogen Novex Mini‐Cell X Cell SureLock (Life Technologies Corporation). Transfers were carried out in 0.5× TBE at 450 mA for 45 min, with the surrounding outer minicell volume packed with ice and filled with water to cool the blot module. Biotin labeled probes were detected on transfer membranes using the ThermoScientific Chemiluminescent Nucleic Acid Detection Module Kit (part number 89880, ThermoScientific) according to manufacturer's instructions.

## 3 RESULTS

### 3.1 *rex* deletion and bioinformatic prediction of the rex regulon in *C. bescii*

Two *rex* deletion mutants were generated for this study; one using strain JWCB005 (Chung et al., 2013) as the genetic parent strain, and the other, using the ethanol‐producing strain JWCB032 (Chung et al., 2014) as the parent strain. Predicted promoter regions in the *C. bescii* genome (taken to be within the 300 bp region upstream of all predicted open reading frames) were analyzed for the presence of a putative Rex transcription factor‐binding site using a *C. saccharolyticus* Rex consensus sequence (Bielen et al., 2013). A total of 63 possible binding sites were identified in the *C. bescii* genome (Table S1) and scored based on their homology to the predicted *C. saccharolyticus* consensus Rex‐binding site sequence (Bielen et al., 2013; Novichkov et al., 2013).

### 3.2 Expression profiling of JWCB005Δ*rex* and selecting transcription factor‐binding sites for in vitro verification

Differential transcript expression was conducted with strain JWCB005Δ*rex* and its parent strain JWCB005 to identify genes and transcriptional units under Rex control. Samples were collected at early, mid, and late log phase, (Figure S2a). An average of 83.2% of RNA‐seq reads that passed quality assurance aligned uniquely to the *C. bescii* genome (S.D. 1.3%), with an average per‐sample genome coverage of 276X (S.D. 28). Increased expression during at least one timepoint in JWCB005Δ*rex* relative to its parent strain was observed for 15 genes, while 9 showed decreased expression (Table S2). A summary of genes identified as being likely members of the Rex regulon in *C. bescii*, identified through differential expression, previous, as well as our own de novo‐binding site predictions, and their corresponding putative Rex‐binding sites were tested by electromobility shift assay (Table 2).

### 3.3 Electromobility shift assays to test rex binding to predicted *C. bescii* binding sites

**Figure 1:**
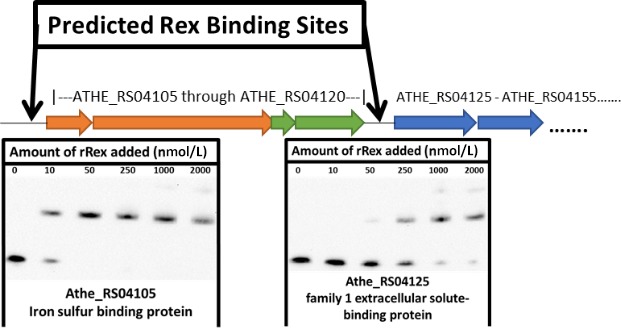
Proposed model of Rex repression supported by electromobility shift assays of binding sites identified upstream of putative transcriptional units associated with a poorly annotated, though highly expressed, oxidoreductase gene. Thes results suggest Rex represses a vaguely annotated, though highly expressed, tungstate‐containing oxidoreductase gene (Scott et al., 2015). rRex Binding upstream of ATHE_RS04125 was found to have a K_d_ much higher than other Rex‐binding sites, suggesting Rex does not bind to this site in vivo. Figure adapted from (Scott et al., 2015). Gene color representations are as represented in (Scott et al., 2015); Blue – tungstate transport, Green – pyranopterin biosynthesis, Orange ‐ ferredoxin and ‘XOR’

**Figure 2:**
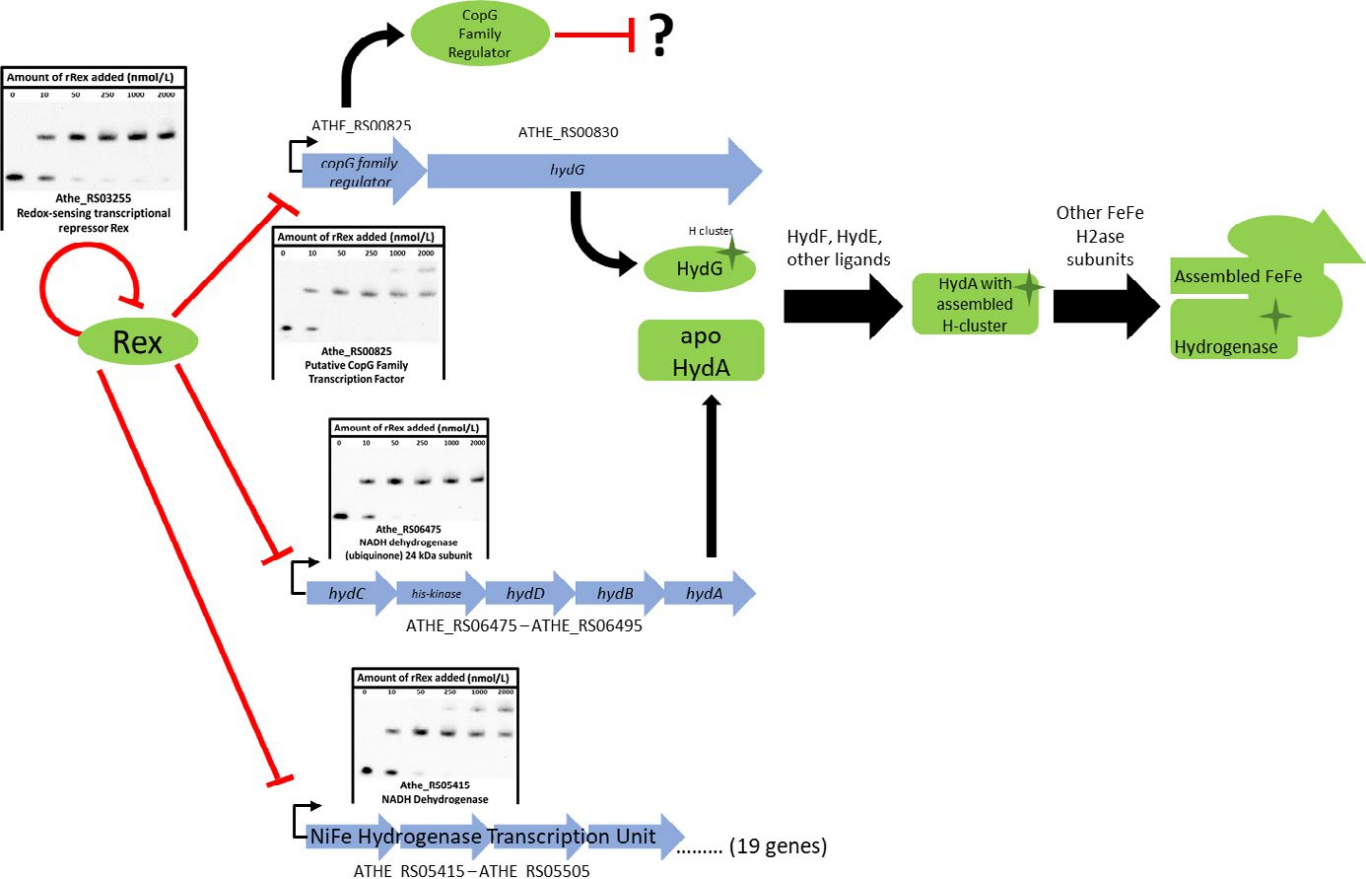
Proposed models of Rex repression supported by electromobility shift assays of Rex‐binding sites upstream of hydrogenase genes in *C. bescii*. These results suggest Rex represses FeFe hydrogenase structural genes and *hydG*, a maturase necessary for active site assembly in FeFe hydrogenases. Rex represses expression of NiFe hydrogenase structural genes. Rex also autoregulates itself. Genomic arrangement of *hyd* and *ech* genes inferred from homology to putative transcriptional units identified in *C. saccharolyticus* (Van de Werken et al., 2008) and predicted transcriptional units identified, using the DOOR prokaryote operon database (Mao et al., 2014). Hyd subunit assembly scheme adapted from (Kuchenreuther et al., 2014)

**Figure 3:**
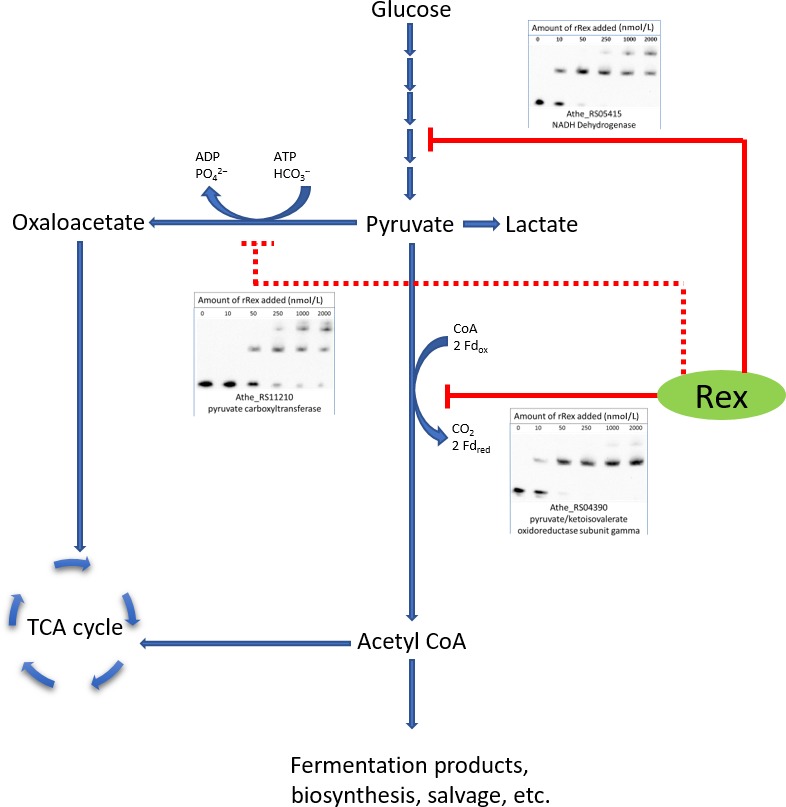
Proposed models of Rex repression supported by electromobility shift assays of binding sites upstream of central glycolytic genes. These results suggest Rex represses expression of ferredoxin‐dependent pyruvate/ketoisovalerate oxidoreductase (Solid red line). Rex transcriptional control of pyruvate carboxyltransferase (ATHE_RS11210, also annotated as oxaloacetate decarboxylase), though remains doubtful because of the relatively high K_d_ observed in vitro (dashed red line)

**Figure 4:**
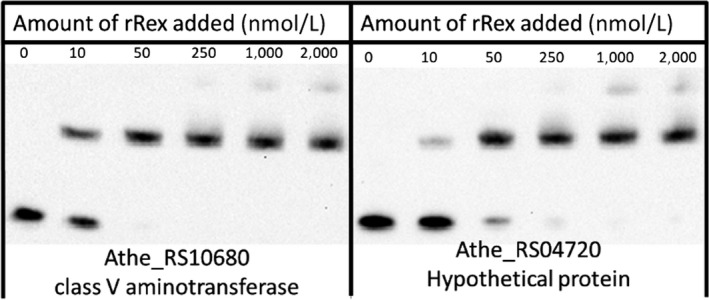
Electromobility shift assays of other predicted Rex‐binding sites whose role in redox metabolism, and *C. bescii* metabolism in general, is not well understood. Rex regulates expression of ATHE_RS10860, a class V aminotransferase as well as ATHE_RS04720, annotated as a hypothetical protein

**Figure 5:**
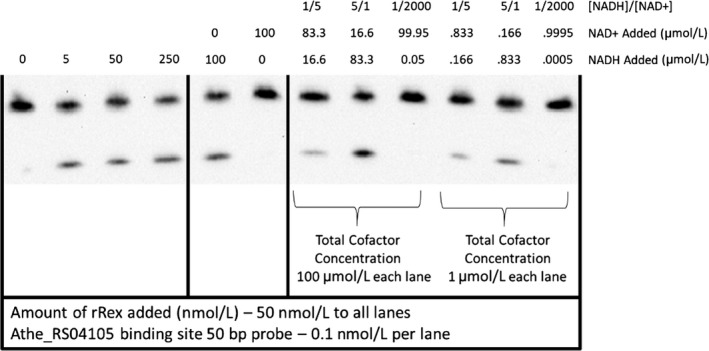
Electromobility shift assay showing DNA binding by Rex is disrupted by NADH and is sensitive to NADH/NAD ^+^ ratio across cofactor pool concentrations of 1–100 μmol/L

A series of operator sites that displayed an in vitro K_d_ between 10 and 50 nmol/L were identified, similar to other in vitro Rex‐binding studies in other Gram‐positive organisms (Brekasis & Paget, 2003; Wang et al., 2008; Zhang et al., 2014). Sequence specificity and NADH cofactor‐binding specificity of in vitro binding reactions were also verified (Figures S4, S5, and 5). Furthermore, operator sites giving these K_d_ values are those located in the promoter regions of genes shown to be conserved members of the Rex regulon (Ravcheev et al., 2012). Four unique *Caldicellulosiruptor* Rex‐binding sites were identified in the promoter regions upstream of the genes ATHE_RS00825, ATHE_RS04105, ATHE_RS04720, and ATHE_RS10680. These genes comprise regulon members for Rex in *Caldicellulosiruptor* not previously identified (Figures 1, 2, 3, 4, 5).

### 3.4 Ethanol productivity of a *rex* deletion in an ethanol producing strain of *C. bescii*

**Figure 6:**
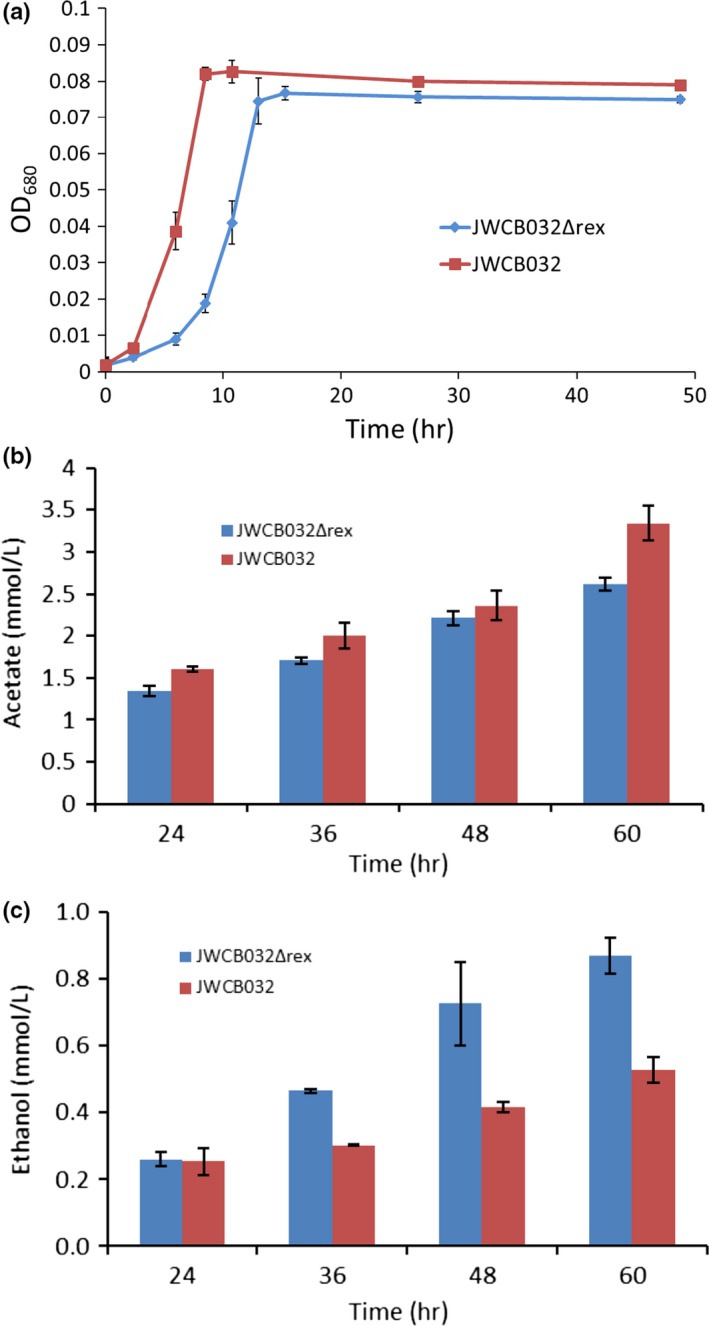
(a) Growth of ethanol producing JWCB032 and JWCB032Δ*rex* conducted in media containing 1/10th of typical concentration of ammonia. (b) Acetate and (c) ethanol produced by strains JWCB032Δ*rex* and JWCB032 showing a shift away from acetate and toward ethanol production after 36 hr of fermentation. Error bars represent one standard deviation of two culture replicates. Points on line plots and values indicated in bar plots are mean values of two biological replicates

To test the direct impact of the Rex protein on ethanol production in *C. bescii*, we deleted the *rex* gene in an ethanol producing strain, JWCB032 (Chung et al., 2014). JWCB032Δ*rex* showed no differences in growth profile or fermentation products relative to its parent after 48 hr when grown in LOD media (Figure S6). However, phenotypic differences were observed when these two strains were grown in LOD media augmented with 1/10th the typical amount of ammonium chloride (0.467 mmol/L), the only source of soluble reduced nitrogen in LOD media (Farkas et al., 2013), making this a nitrogen‐limited growth condition. *C. bescii* actively grows for only 10–12 hr when grown in this condition (Figure 6a). Both strains grew to similar turbidity under this growth condition, with the *rex* mutant exhibiting a lag phase about 5 hr longer than its parent strain (Figure 6). Differences in fermentation product profile appear after active growth ceases (>36 hr, Figure 6b). The *rex* knockout strain produced 54% more ethanol after 36 hr of fermentation than its parent strain (Figure 6), accounting for a 0.16 mmol/L difference in final ethanol concentration between the two strains. The two strains produced similar amounts of acetate after 36 hr of fermentation. The relative difference in the amount of ethanol produced in JWCB032Δ*rex* relative to JWCB032 increased through 60 hr of fermentation.

### 3.5 Metabolite profile differences observed in ethanol producing rex‐deficient *C. bescii*

**Figure 7:**
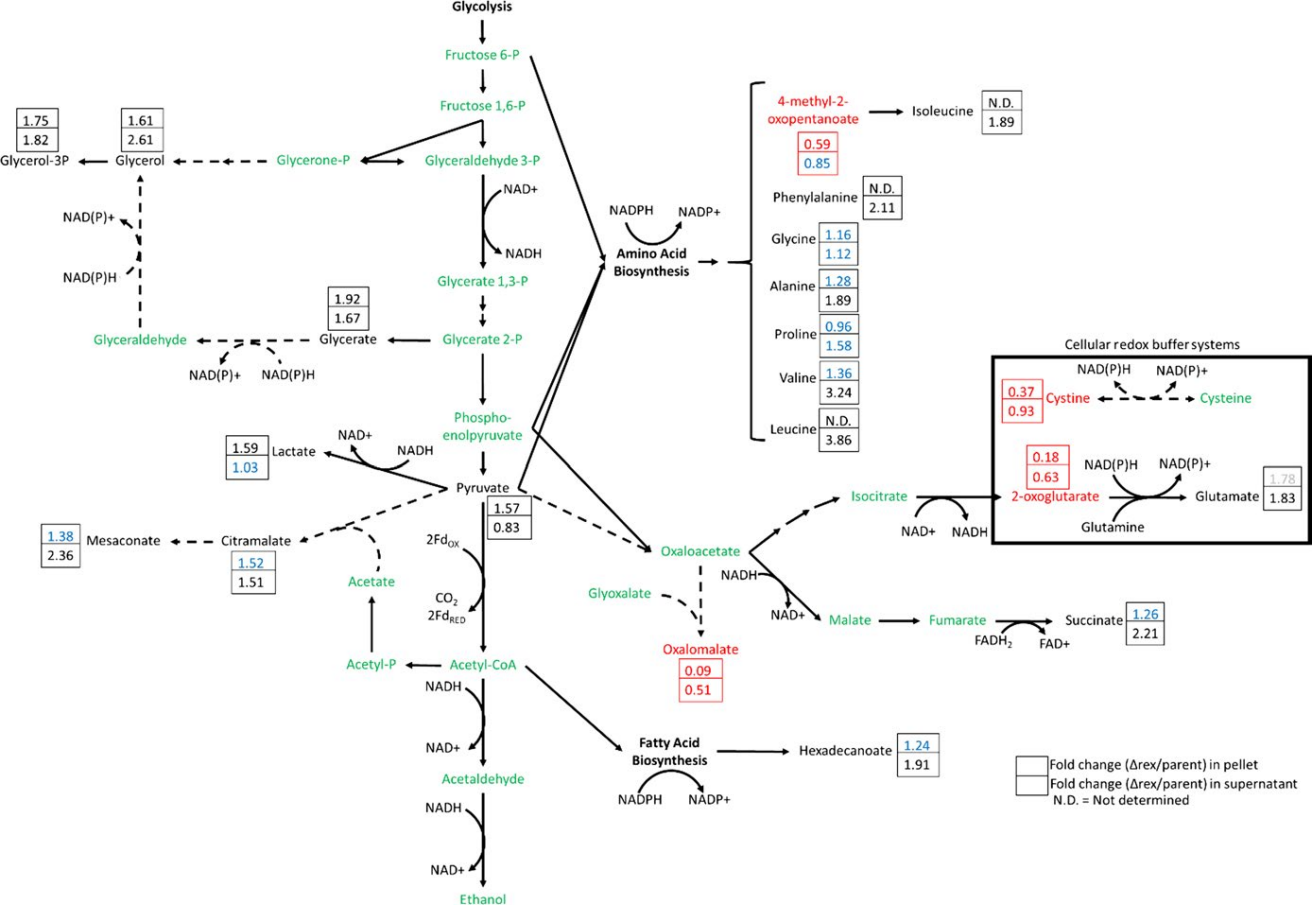
Differential metabolomic comparison of JWCB032Δ*rex* and its parent strain JWCB032. Metabolites indicate a metabolic shift toward reductive metabolic reactions in JWCB032Δ*rex*, indicative of more reduced intracellular redox status and possibly being driven by the accumulation of pyruvate. Solid lines indicate reactions annotated in KEGG for *C. bescii*, while dashed black lines indicate reactions not annotated in the *C. bescii* KEGG database entry. Values indicated in boxes are relative abundance ratios, computed as signal intensities identified in rex‐deficient mutant strain relative to that in the genetic parent strains. Metabolites labeled in green were either not detected or their fold‐change differences were not statistically significant. Red colored metabolites showed significant decreased metabolites levels in JWCB032Δ*rex* (relative to its parent strain JWCB032) while black colored metabolites showed significantly increased metabolite levels. Black numbers indicate values that were found to be statistically significant, while blue numbers are not statistically significant. Significance was determined as *p* < .05 from a two‐tailed students *t*‐test (*n* = 4 biological replicates, equal variance assumed). Twelve replicate cultures were collected, and material from three cultures were combined to make one analytical replicate and four replicates per strain. Pellets and supernatants of four analytical replicates of each strain were thus analyzed, separately

To investigate ethanol production differences between the two strains under nitrogen‐limiting conditions, intracellular, and extracellular metabolomic profiles were generated after 36 hr of fermentation (Figure 7, Table S3), when differences in ethanol concentrations were previously observed to be prominent (Figure 6). Succinate and 2‐oxoglutarate, two TCA cycle metabolites, show a shift in TCA cycle carbon flux toward succinate production in JWCB032Δ*rex*. The relative intracellular abundance of 2‐oxoglutarate in JWCB032Δ*rex* was only 18% of that of strain JWCB032, while the corresponding relative intracellular concentrations of succinate were 125% higher in JWCB032Δ*rex*. Lactate, pyruvate, glycerol/glycerol‐3P, and hexadecanoate were all found to be at least 124% more abundant in JWCB032Δ*rex*. The relative abundance of many amino acids was increased either intracellularly or in the supernatant in JWCB032Δ*rex*. Mesaconate and citramalate were found to be at least 138% more abundant in JWCB032Δ*rex* in both the supernatant and intracellularly, while oxalomalate was found to be less abundant in both. Cystine was found in decreased abundance in JWCB032Δ*rex*, both intracellularly (37% of parent strain) and in the supernatant (93% of parent strain).

## 4 DISCUSSION

### 4.1 Expanding the *rex* regulon in *C. bescii*

The Rex regulon of the close *C. bescii* relative, *Caldicellulosiruptor saccharolyticus*, has been curated in the RegPrecise database (Novichkov et al., 2013). RegPrecise predicts six Rex‐binding sites in *C. saccharolyticus*. Two of these sites are located upstream of *adhA* genes (Csac_0622 and Csac_0407) of which *C. bescii* does not have a homolog of. Homologs of the remaining four predicted Rex operator sites predicted in *C. saccharolyticus* were found on the *C. bescii* genome (Table 2). A study of gene expression in *C. saccharolyticus* under hydrogen sparging (Bielen et al., 2013) found evidence to extend the putative Rex regulon of *C. saccharolyticus* beyond those genes predicted in RegPrecise. Our study validates Rex regulatory control of these previously predicted regulon members, as well as novel members of the Rex regulon not previously predicted; the HydG hydrogenase maturation factor collocated in a putative operon with a CopG family transcription factor, the ‘XOR’ gene (a poorly annotated oxidoreductase) (Scott et al., 2015), an unannotated highly expressed oxidoreductase gene, and a class V aminotransferase.

HydG is responsible for synthesizing a di‐iron precursor to the H‐cluster active site of the FeFe hydrogenase (Kuchenreuther et al., 2014). As the presence or absence of HydG protein can effectively regulate assembly of functional FeFe hydrogenase by modulating correct active site assembly (Biswas et al., 2015), and the FeFe hydrogenase is a main hydrogen generation route in *C. bescii* (Cha et al., 2015), its regulation by Rex is consistent and expected. The regulon of this CopG transcription factor remains unknown and unpredicted.

Rex regulation of the ‘XOR’ gene is also to be expected given its presumed importance to redox metabolism in *C. bescii* (Scott et al., 2015). While Rex is only known to sense the redox potential of the NADH/NAD^+^ redox couple within the cell, it regulates redox metabolic reactions whose cofactors stretch beyond just this couple, exemplified in *C. bescii* by Rex regulation of the ferredoxin‐dependent NiFe membrane bound hydrogenase (Figure 2) and of a likely ferredoxin‐dependent pyruvate:ferredoxin oxidoreductase (Figure 3). While the specific function of this gene remains unknown, its relatively high expression level, unique reliance on tungsten, and coordinated expression with tungsten assimilation genes and a ferredoxin suggest its importance to redox metabolism in *C. bescii*.

Rex regulatory control of a class V aminotransferase in *C. bescii* is not well understood. A serine‐pyruvate aminotransferase was identified as part of the Rex regulon of five genomes from *Thermotogales*, constituting an NADH‐dependent step of a serine utilization pathway (Ravcheev et al., 2012). This Rex regulated class V aminotransferase in *C. bescii* is annotated as an alanine‐glyoxylate aminotransferase and no reference is given to its redox dependence. This gene is also collocated in a putative transcriptional unit with an NADH dependent 3‐phosphoglycerate dehydrogenase, also a biochemical step in serine biosynthesis.

### 4.2 Ethanol‐producing *rex*‐deficient *C. bescii* produces more ethanol under nitrogen‐limiting conditions which extends fermentation

A *rex* deletion strain was generated, using *C. bescii* strain JWCB032 (Chung et al., 2014) as the parent to investigate the effect Rex may have on ethanol production and overall redox metabolism. Relative differences in ethanol synthesis were observed in a *rex‐*deficient mutant strain when the cells were grown in nitrogen‐limiting conditions (Figure 6), though not in replete media, and only after 36 hr of fermentation. Under replete conditions, the pH of batch cultures of *C. bescii* drop to ~4.5 from an initial pH of 7.2 (data not shown) due mainly to the production of acetic acid. Growth and fermentation are not observed at or below these pH values (data not shown). Growing the cells in nitrogen‐limiting conditions restricts active growth, the total amount of cell biomass synthesized, and acts to limit the total amount of acetate that is generated (as seen by comparing nitrogen limited growth and fermentation products in Figure 6 and replete fermentation product production in Figure S6), allowing fermentation to continue long after active growth has ceased. A shift was observed in whole‐cell redox state in *C. saccharolyticus* as it entered the stationary phase, as indicated by an increase in lactate production and lower response when harvested cells were subjected to a poised amperometric cell (Kostesha, Willquist, Emneus, & van Niel, 2011). This indicates redox‐mediated end‐product shifting is occurring as cells enter stationary phase, likely favorable conditions for ethanol production as the exogenous AdhE of JWCB032 and native lactate dehydrogenase both rely on NADH to generate their respective products. *C. cellulolyticum* grown in nitrogen‐limited chemostat culture similarly showed an increase in fermentative flux toward ethanol as dilution rates increased (Desvaux & Petitdemange, 2001). Ethanol generation by heterologously expressed AdhE protein expressed in strain JWCB032 has been shown to be dependent on redox conditions in the native host. Perturbing redox metabolism in *C. thermocellum*, the native host of this AdhE enzyme, either through genetic modification or altering growth conditions, has predictably resulted in altered levels of the formation of ethanol and other reduced fermentation products (Biswas et al., 2015; Brown et al., 2011; Rydzak, Levin, Cicek, & Sparling, 2011; Sander et al., 2015). It is likely this AdhE enzyme is similarly sensitive to redox conditions when expressed in *C. bescii*.

Another possible explanation for elevated levels of ethanol in JWCB032Δ*rex* under nitrogen limitation would be an interaction between the *rex* regulator and nitrogen metabolism in *C. bescii,* which could be augmented in the JWCB032Δ*rex* strain deficient in this regulator. Metabolic and expression coupling between nitrogen metabolism and ethanol production has been studied in mutant strains of *C. thermocellum* (Rydzak et al., 2017), though no regulatory link involving Rex was found. No known link between nitrogen metabolism and ethanol production involving Rex in *C. bescii* is known to exist either.

To investigate why JWCB032Δ*rex* produced more ethanol than its parent strain, intracellular, and extracellular metabolomics was conducted on this strain and its parent, JWCB032. Most metabolites found in increased abundance in strain JWCB032Δ*rex* require reduction reactions (with reductant being provided by NADH and NADPH in most cases) for their synthesis. Apart from the glycerol and glycerol‐3P, inferred metabolic pathways (Kanehisa, Sato, Kawashima, Furumichi, & Tanabe, 2016) responsible for the synthesis of metabolites found in differential abundance originate at the pyruvate metabolic pathway node (Figure 7). The accumulation of pyruvate and the redox‐state reduction of intracellular redox pools may support the synthesis of overflow metabolites.

### 4.3 Redox buffering systems

Increased concentrations of components of two known redox buffer systems were observed in JWCB032Δ*rex* cells; the cysteine–cystine couple (Banerjee, 2012) and the 2‐oxoglutarate‐glutamate couple (Ballester‐Tomás, Randez‐Gil, Pérez‐Torrado, & Prieto, 2015; Mailloux et al., 2009). The reactions of both couples involve the interchange of one molecule of NAD(P)(H) and 2 electrons. The oxidized component of each couple was found in decreased concentration in JWCB032Δ*rex*, suggesting that these redox buffer systems are responding to more reduced redox conditions relative to those of strain JWCB032 by reducing the pools of these molecules. Cysteine is known to be one of the most reducing of the known cellular redox buffer systems (Banerjee, 2012). Free cysteine has been shown to reduce iron in *E. coli*, generating hydroxyl radicals and causing DNA damage, and this may be an evolutionary reason intracellular free cysteine levels are kept low in cells (Park & Imlay, 2003). Components of other cellular redox buffer systems were not detected in our metabolomic study of JWCB032 and JWCB032Δ*rex*. *C. bescii* redox conditions may be more readily augmented using the cysteine–cystine system rather than other redox buffering systems.

### 4.4 Differentially abundant metabolites in JWCB032Δrex

Metabolites involved in glycerol metabolism, biosynthesis of the fatty acid hexadecanoate, five amino acids (isoleucine, phenylalanine, alanine, valine and leucine), TCA cycle metabolites, ethanol, and acetate were found in increased abundance in JWCB032Δ*rex*. As the genes responsible for biosynthesis of these metabolites were not differentially expressed in strain JWCB005Δ*rex*, we hypothesize that these differences result from metabolite‐driven flux. The metabolites glycerate, glycerol, and glycerol 3‐phosphate were found in relatively increased abundance in strain JWCB032Δ*rex*, and two reactions involved in the synthesis of these metabolites (glyceraldehyde dehydrogenase and glycerol dehydrogenase) are redox dependent. There are no genes annotated in the *C. bescii* KEGG database entry (Kanehisa et al., 2016) that encode catalytic enzymes for these reactions, nor are there any genes annotated with these functions in the recently re‐annotated RefSeq annotations for the *C. bescii* DSM 6725 genome, though the presence of these molecules indicates these functions must be present in vivo in *C. bescii*. It is worth noting that *C. bescii* cannot grow in media containing glycerol as a sole source of carbon (Hamilton‐Brehm et al., 2010). Increased abundance of amino acids, particularly branched chain amino acids, was also observed as products of overflow metabolism of *C. thermocellum* (Biswas et al., 2017; Holwerda et al., 2014) in response to genetically induced redox perturbations and increased substrate loading. Metabolic flux of the TCA cycle and associated reactions was also redistributed toward reductive reactions, resulting in increased supernatant concentrations of succinate and glutamate and decreased supernatant and intracellular concentrations of oxalomalate and 2‐oxoglutarate. Succinate production was increased 60% in *E. coli* by altering redox metabolism (Singh, Cher Soh, Hatzimanikatis, & Gill, 2011), highlighting the importance intracellular redox can have on the synthesis of succinate and TCA cycle flux.

According to the KEGG functional annotation (Kanehisa et al., 2016) of the *C. bescii* genome, metabolites whose synthesis do not require redox reactions in reaction steps unique to their synthesis are mesaconate, citramalate, oxalomalate, and acetate. Acetate and oxalomalate are competing pathways to metabolites whose synthesis pathways do depend on redox reactions, and the relatively decreased amount of these metabolites may be due to redox‐driven flux through these competing pathways. Mesaconate can be synthesized from pyruvate or glutamate. Citramalate and mesaconate production are sequential metabolic steps, and because citramalate is also found in increased abundance, mesaconate is likely synthesized from pyruvate rather than from glutamate. KEGG does not annotate/assign genes for mesaconate synthesis from pyruvate or glutamate in *C. bescii*, though the most recent RefSeq annotation of the *C. bescii* DSM 6725 genome does contain an annotation for a gene encoding a citramalate synthase (ATHE_RS02505). No gene encoding a mesaconate hydratase, the enzyme synthesizing mesaconate from citramalate, could be identified. Neither of the two genes necessary for mesaconate synthesis from glutamate are annotated and protein BLAST homology searches for methylaspartate ammonia‐lyase from *E. coli* and *Aspergillus oryzae* returned no genes with significant similarity. This further suggests mesaconate is being generated from citramalate as an intermediate and as an overflow metabolism product resulting from the accumulation of pyruvate.

### 4.5 Intracellular redox conditions and pyruvate accumulation possibly driving metabolite differences

Many reactions synthesizing differentially abundant metabolites are [NADH]/[NAD^+^] dependent, and the observation of cellular redox buffer systems being active, further supports the hypothesis that metabolite differences observed in JWCB032Δ*rex* stem, in part, from redox‐driven flux differences. Redox‐driven overflow metabolism is observed in two other CBP organisms, *Clostridium thermocellum* (Holwerda et al., 2014) and *Clostridium cellulolyticum* (Guedon, Payot, Desvaux, & Petitdemange, 1999). Excess‐free amino acids are found in the supernatant of strains of *C. thermocellum* whose redox metabolism has been perturbed (Holwerda et al., 2014), and strains of *C. cellulolyticum* grown at high dilution rates (Guedon et al., 1999). Upon being challenged with these increased reductive loads, pyruvate is found in the supernatant of both species and both species synthesize lactate (Guedon et al., 1999; Holwerda et al., 2014). *C. cellulolyticum* also shunts carbon at an earlier glycolytic node (Glucose 1‐phosphate → Glucose 6‐phosphate) toward glycogen and exopolysaccharide synthesis in response to increasing substrate loads (Guedon, Desvaux, & Petitdemange, 2000). Pyruvate accumulation and subsequent overflow metabolism has been observed and studied in detail in *C. thermocellum* (Deng et al., 2013; Holwerda et al., 2014; Olson et al., 2017; Thompson et al., 2015). Accumulation of formate and hydrogen in *C. thermocellum* collectively restrict the reoxidation of ferredoxin and limit its availability for pyruvate:ferredoxin oxidoreductase‐enabled conversion of pyruvate to acetyl‐CoA, causing accumulation of pyruvate and pyruvate‐derived overflow metabolism products (Holwerda et al., 2014; Thompson & Trinh, 2017; Thompson et al., 2015). *C. cellulolyticum* growth was shown to be inhibited by high concentrations of NADH, which inhibits glyceraldehyde 3‐phosphate dehydrogenase activity and limits glycolytic flux (Payot, Guedon, Cailliez, Gelhaye, & Petitdemange, 1998). Substantial overflow metabolism and increased ethanol synthesis was observed at relatively lower [NADH]/[NAD^+^] ratios in *C. cellulolyticum* (Guedon et al., 1999), as to where strains of *C. thermocellum* demonstrating the most overflow metabolism and highest ethanol yields generally exhibit relatively higher [NADH]/[NAD^+^] ratios (Beri, Olson, Holwerda, & Lynd, 2016), suggesting [NADH]/[NAD^+^] ratio alone may not always determine flux directionality and flux toward overflow products. Overflow metabolism may be similarly metabolite and/or redox driven in *C. bescii*, though direct measurement of intracellular redox conditions and/or validated metabolic modeling would further qualify this hypothesis.

## 5 CONCLUSIONS

In studying the *rex* gene in *C. bescii*, we have found novel members of the Rex regulon not previously described. These include the ‘XOR’ gene (Scott et al., 2015), an oxidoreductase gene that is highly expressed in *C. bescii*, a class V aminotransferase (ATHE_RS10680), a hypothetical protein (ATHE_RS04720), and the *hydG* gene contained in a putative transcriptional unit along with a CopG family transcription factor. The functional contribution of these genes to redox homeostasis, the primary function of the Rex protein, remains unknown. A *rex* deletion mutant strain heterologously expressing a bifunctional alcohol dehydrogenase gene produced 54% more ethanol (a 0.16 mmol/L increase in final titer) when fermentation continued for 36 hr (while limited for nitrogen). Metabolomic profiling shows differential abundance of reduced products and the shift of two known redox buffering systems toward their reduced counterparts, suggesting the elimination of the *rex* gene leads to a more reduced intracellular redox environment in the stationary phase that in turn drives increased production of ethanol and other overflow metabolites.

## ACCESSION NUMBERS

Raw reads, processed data and experiment metadata were submitted to the NCBI Gene Expression Omnibus (GEO) under accession number GSE102041.

## CONFLICT OF INTEREST

None declared.
