# Supplementary material for: Rex in Caldicellulosiruptor bescii: Novel regulon members and its effect on the production of ethanol and overflow metabolites
Source: Microbiologyopen. 2018 May 23;8(2):e00639. doi: 10.1002/mbo3.639 (PMC6391272; doi:10.1002/mbo3.639)
Supplement: Supplementary file 1 [file MBO3-8-e00639-s001.docx]

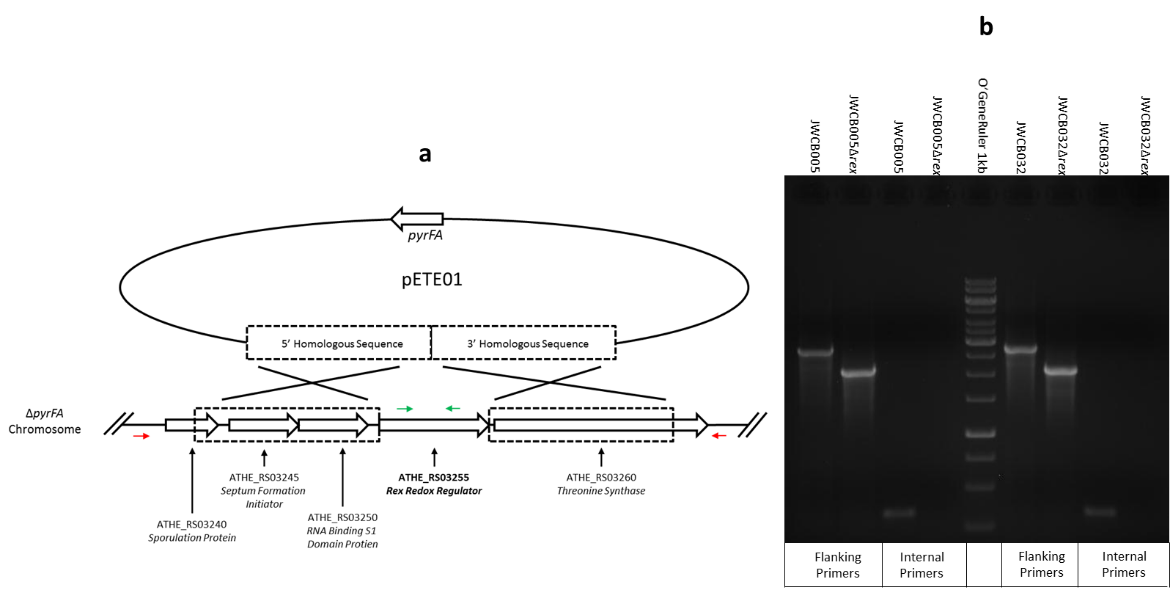


Figure S1. (a) Chromosomal integration and recombination scheme for constructing Δ*rex* mutants of JWCB005 and JWBCB032. Red arrows distal to homology region indicate primer binding sites for flanking primers used to screen knockout strains. Green arrows inside ATHE_RS03255 coding DNA sequence indicate primers binding internal to *rex* gene used to screen for the presence of *rex* coding sequence DNA. (b) PCR confirmation of Δ*rex* in strains JWCB005 and JWCB032 using primers which anneal within the deleted CDS (internal primers) and primers which anneal outside of the 5’ and 3’ flanking regions of homology (flanking primers). Expected *rex* wt amplicon using flanking primers is 2825 bp and expected Δ*rex* amplicon using flanking primers is 2171 bp. Expected amplicon length of PCR product using internal primers is 348 bp.


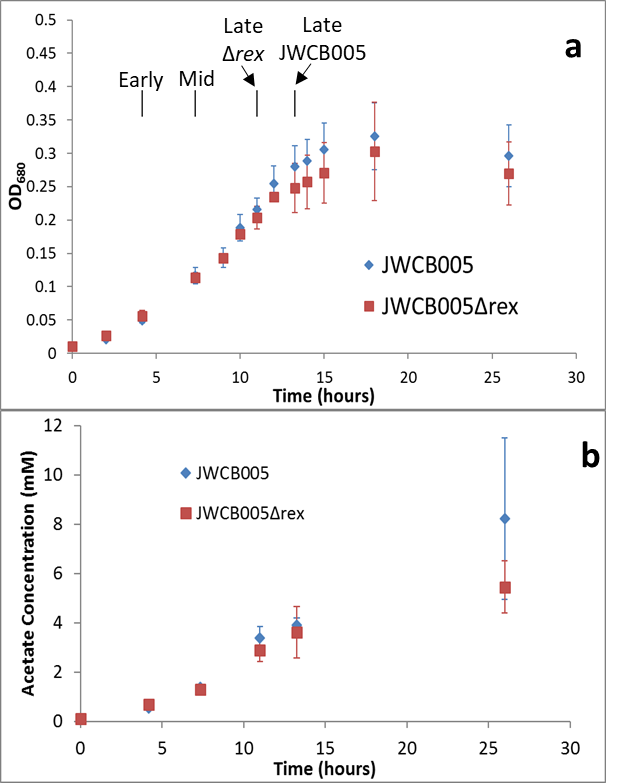


Figure S2. (a) Growth profile and (b) supernatant acetate concentration of JWCB005Δ*rex* strain and parent strain JWCB005. Labeled marks indicate times when cell aliquots were collected for expression profiling. No lactate was detected during growth.


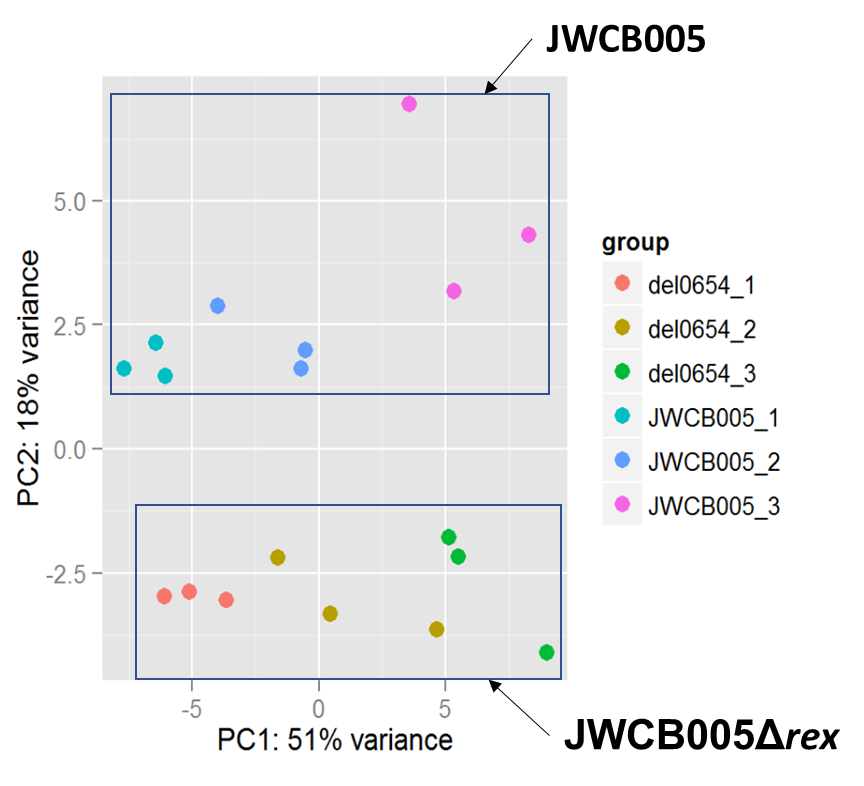


Figure S3. Principal component analysis of normalized mapped RNA-seq read-counts from strains JWCB005 and JWCB005Δ*rex*.


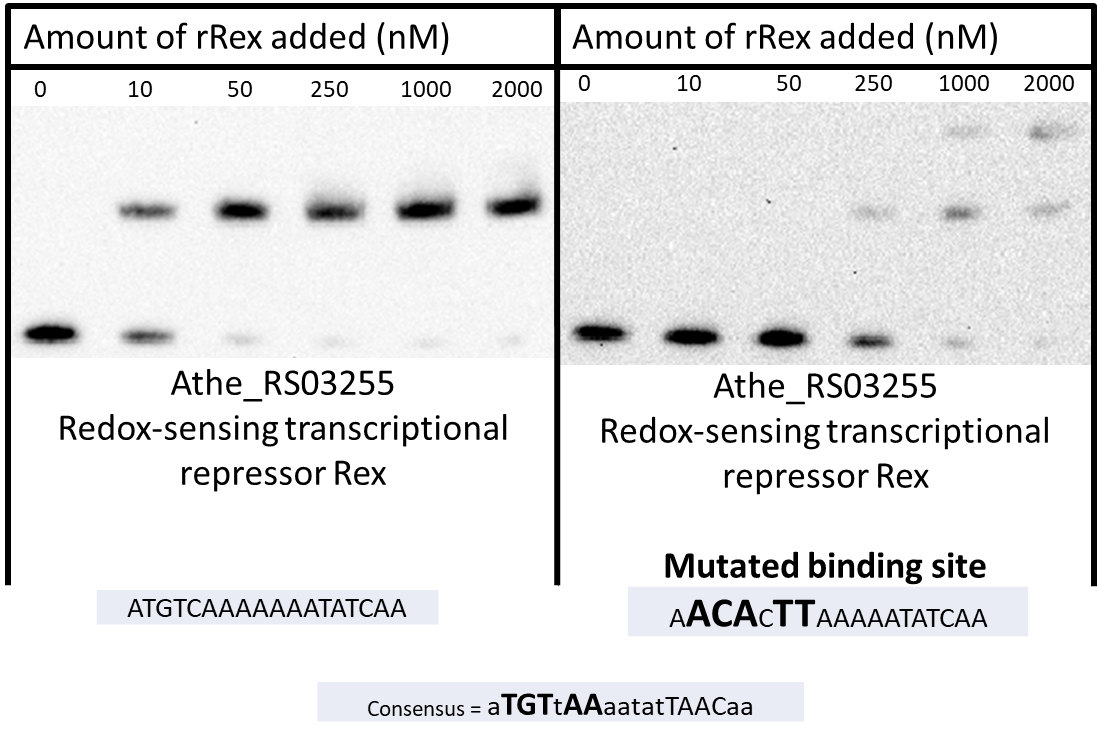


Figure S4. Electromobility shift assay using ATHE_RS03255 probe containing a mutated Rex binding site showing *in vitro* EMSA assay is sequence specific for Rex operator sites.


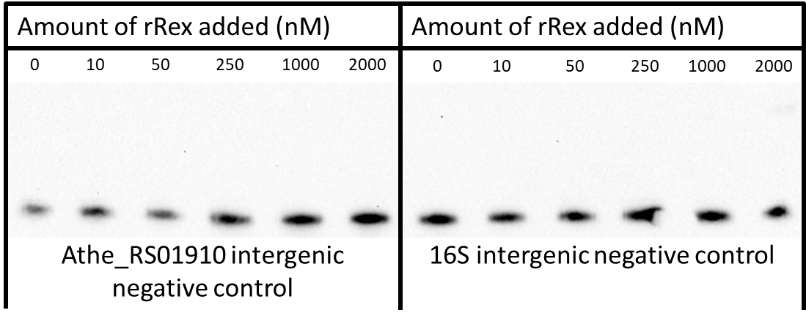


Figure S5. Electromobility shift assay reactions using probes containing non-homologous sequence to Rex binding sites showing Rex binding is sequence specific for previously predicted operator sites.


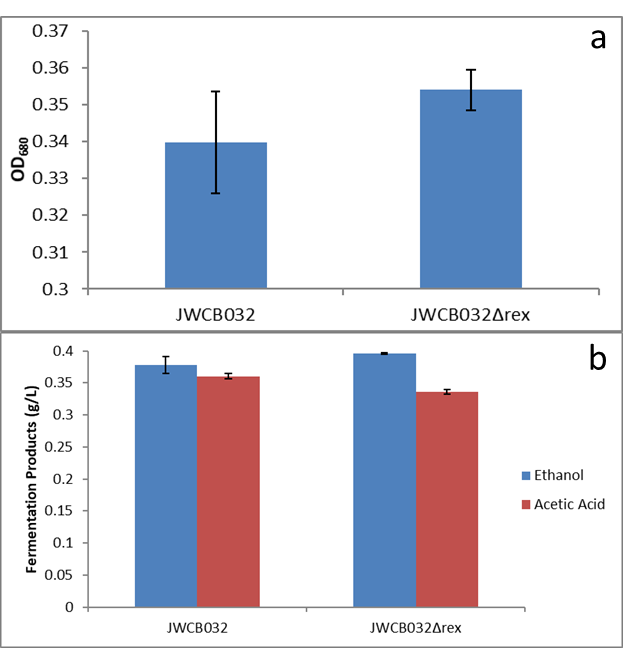


Figure S6: (a) Cell growth (as measeured by OD_680_) and (b) fermentation products of strains JWCB032 and JWCB032Δ*rex* after 48 hours of growth in LOD media.
